# Role of Transport Polarity in Transient Electroluminescence of Two-Dimensional TMDC Semiconductors

**DOI:** 10.3390/nano16130827

**Published:** 2026-07-06

**Authors:** Xin Yang, Kai Liu, Rui Huang, Zixing Zou, Chenguang Zhu, Feng Jiang, Ying Chen, Yushuang Zhang, Lei Shan

**Affiliations:** 1 State Key Laboratory of Opto-Electronic Information Acquisition and Protection Technology, Information Materials and Intelligent Sensing Laboratory of Anhui Province, Institutes of Physical Science and Information Technology, School of Physics, Anhui University, Hefei 230601, China; q125111003@stu.ahu.edu.cn (K.L.); b124308016@stu.ahu.edu.cn (R.H.); 2 School of Electronic Information and Electrical Engineering, Changsha University, Changsha 410022, China; z20220929@ccsu.edu.cn; 3 College of Materials Science and Engineering, Hunan University, Changsha 410022, China; chen_zhu@hnu.edu.cn; 4 State Key Laboratory for Advanced Metals and Materials, University of Science and Technology, Beijing 100083, China; jiangfeng@ustb.edu.cn; 5 State Key Laboratory of Pulsed Power Laser Technology, Key Laboratory of Electronic Restriction of Anhui Province, Advanced Laser Technology Laboratory of Anhui Province, College of Electronic Engineering of NUDT, National University of Defense Technology, Hefei 230037, China

**Keywords:** two-dimensional materials, transition metal dichalcogenides, electroluminescence, transport polarity, carrier tunneling

## Abstract

Two-dimensional transient electroluminescent devices have attracted considerable attention owing to their simple device architecture and reduced contact-barrier dependence. However, the influence of semiconductor transport polarity on transient electroluminescence (EL) remains unclear. Here, we compare four representative transition metal dichalcogenide (TMDC) semiconductors with different transport polarities and find that ambipolar WSe_2_ exhibits a stronger transient EL signal under identical driving conditions, a trend that cannot be explained by relative photoluminescence quantum yield (PLQY) alone. Transfer characteristics and gate-modulated photoluminescence (PL) measurements were further used to analyze the gate-dependent carrier doping states and the local spectral response associated with interfacial carrier modulation near the metal/TMDC interface during abrupt gate-voltage switching. Based on these results, we propose a possible physical picture in which ambipolar WSe_2_ is more likely to form a transient interfacial electron–hole distribution favorable for electron–hole radiative recombination, whereas predominantly n-type materials tend to form electron-rich interfacial carrier states. These findings suggest that semiconductor transport polarity is an important material factor for designing low-dimensional transient electroluminescent devices.

## 1. Introduction

On-chip light sources, which encode electrical signals into optical information, are indispensable components for optical interconnects and Si-based photonic integrated circuits (PICs) [1,2,3,4,5,6,7,8,9,10,11,12,13]. Two-dimensional transition metal dichalcogenides (TMDCs), emerging over the past decade, offer a promising material platform for such light sources owing to their exceptional properties, including atomic-scale thickness, direct bandgaps in the monolayer limit, and lattice-matching-free heterointegration with traditional CMOS technology platforms [11,14,15,16,17,18,19,20]. TMDC-based light-emitting devices mainly involve several device architectures, including Schottky-junction devices [21], homo/heterogeneous p–n diodes [22,23,24,25,26], tunnel-junction devices [27,28,29,30,31,32], and transient metal–insulator–semiconductor (MIS) devices [33,34,35,36,37]. Among these device architectures, transient MIS electroluminescent devices provide a promising route for achieving efficient electroluminescence in low-dimensional semiconductors, owing to their simple device architecture and the reduced dependence of carrier injection on the Schottky barrier height. Previous studies mainly attributed the transient electroluminescence (EL) intensity to the amplitude and frequency of the AC driving voltage, as well as the intrinsic quantum yield of the emitting layer [34,38,39,40,41]. These parameters are related to transient band bending at the metal–semiconductor interface, the number of transient EL pulses generated per unit time, and the conversion efficiency from electron–hole pairs to radiative photons, respectively. Because transient EL originates from radiative recombination of electron–hole near the metal–semiconductor interface, the interfacial electron–hole population established before gate-voltage switching is expected to influence the subsequent transient recombination process. Semiconductor carrier polarity may be one of the key factors affecting this initial carrier population. However, the role of semiconductor transport polarity in transient EL has not been systematically investigated. Here, by comparing the corrected EL intensities of four representative TMDC semiconductors, we find a clear correlation between transient EL intensity and transport polarity. By combining relative PLQY, transfer characteristics, and gate-modulated PL measurements, we further analyzed the carrier doping states of different materials under gate modulation, as well as the spectral responses associated with interfacial carrier tunneling during abrupt gate-voltage switching. Based on these results, we propose a possible physical picture in which transport polarity may modulate transient EL emission by influencing the interfacial electron–hole distribution after gate-voltage switching. This explanation does not exclude the contributions from contact properties, Schottky barriers, and other material-related factors to the absolute EL intensity, but provides a reasonable basis for understanding the polarity-related emission behavior observed experimentally. These results provide a basis for understanding how semiconductor transport polarity may influence transient EL emission in low-dimensional semiconductors.

## 2. Materials and Methods

Device Fabrication: Monolayer TMDC flakes (WS_2_, WSe_2_, MoS_2_, and MoSe_2_) were mechanically exfoliated from the corresponding bulk crystals (obtained from Nanjing MKNANO Tech, www.mukenano.com, Nanjing, China) using Scotch tape. The exfoliated flakes were first transferred onto a polydimethylsiloxane (PDMS) stamp, and selected monolayer flakes were then deterministically released onto SiO_2_/Si substrates with the aid of an optical microscope and a three-axis micromanipulation stage. This dry-transfer strategy based on a viscoelastic stamp has been widely adopted for the fabrication of two-dimensional material devices because it enables accurate positioning of thin flakes while avoiding liquid-assisted transfer steps. After the transfer of monolayer TMDCs, the substrates were spin-coated with PMMA resist at 1000 rpm for 3 s followed by 4000 rpm for 60 s. Electrode patterns were subsequently defined by electron-beam lithography and developed in the PMMA layer. Metal electrodes (Cr/Au, 20/50 nm) were then deposited by thermal evaporation, followed by lift-off in acetone to remove the resist mask. Electron-beam lithography combined with metal deposition and lift-off is a standard microfabrication route for TMDC-based electronic and optoelectronic devices.

Optical Measurements: Optical spectroscopy measurements were performed using a commercial confocal micro-photoluminescence (μ-PL) system (WITec, alpha-300, WITec GmbH, Ulm, Germany). A 532 nm continuous-wave laser was used as the excitation source for PL measurements of the TMDC samples. The emitted PL signal was collected by the same objective lens and guided into a grating monochromator equipped with a thermoelectrically cooled charge-coupled device (CCD) detector for spectral analysis. All measurements were carried out at room temperature in reflection configuration. Time-dependent PL CCD imaging was performed using a home-built confocal microscope optical system. A 532 nm laser focused by a 100× objective (Olympus MPlanFL N, NA = 0.9, Evident Corporation, Tokyo, Japan) was used to excite the TMDCs, and the PL signal was collected by the same objective. A 590 nm long-pass dichroic mirror was used to filter the excitation laser. Then, the PL signal was guided into a grating monochromator equipped with a thermoelectrically cooled CCD camera (Teledyne, HORIBA Scientific, Piscataway, NJ, USA).

Electrical Measurements: Electrical measurements were carried out in a vacuum probe station under high-vacuum conditions (10^−4^ Pa) at room temperature to minimize the influence of ambient adsorbates and moisture on the electrical characteristics of the TMDC devices. The electrical transport properties were characterized using a semiconductor parameter analyzer (Agilent B1500A, Agilent Technologies, Santa Clara, CA, USA). The heavily doped Si substrate was employed as a global back gate, while the thermally grown SiO_2_ layer served as the gate dielectric. Selected probe needles in a six-probe vacuum probe station were used for device biasing and signal acquisition. Gate-dependent transfer characteristics were obtained by applying bias voltages through the semiconductor parameter analyzer. All measurements were performed at room temperature unless otherwise specified.

Electroluminescence Measurements: For EL measurements, the devices were driven by applying bipolar square-wave gate voltages generated by an arbitrary waveform generator (Tektronix AFG1000 series, Tektronix, Beaverton, OR, USA). The transient EL emission induced by abrupt gate-voltage switching was collected through the microscope objective and analyzed using the same optical spectroscopy setup described above. The waveform amplitude and frequency were adjusted according to the measurement conditions. Unless otherwise specified, all EL measurements were carried out under ambient room-temperature conditions. During the measurements, the metal electrodes were grounded, while the square-wave voltage was applied to the back gate in order to periodically modulate the carrier injection and extraction processes near the metal–semiconductor interface.

## 3. Results

**Figure 1:**
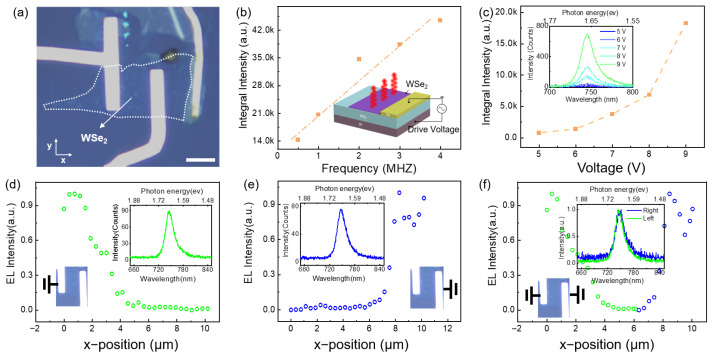
Characterization of the WSe_2_-based transient electroluminescent device. (**a**) Optical image of the WSe_2_-based transient EL device. The scale bar is 10 μm. (**b**) Frequency dependence of the integrated EL intensity. Inset: schematic illustration of the transient EL device structure. (**c**) Square-wave voltage-amplitude dependence of the integrated EL intensity. Inset: EL spectra of the device under different voltage amplitudes. (**d**) Spatial profile of the EL intensity along the x direction when the left electrode in Figure 1a is grounded. The right edge of the left electrode is defined as x = 0. Inset: EL spectrum of WSe_2_ collected near the left electrode. (**e**) Spatial profile of the EL intensity along the x direction when the right electrode in Figure 1a is grounded. Inset: EL spectrum of WSe_2_ collected near the right electrode. (**f**) Spatial profile of the EL intensity along the x direction when both electrodes in Figure 1a are grounded. Inset: normalized EL spectra collected near the left and right electrodes.

**Figure 2:**
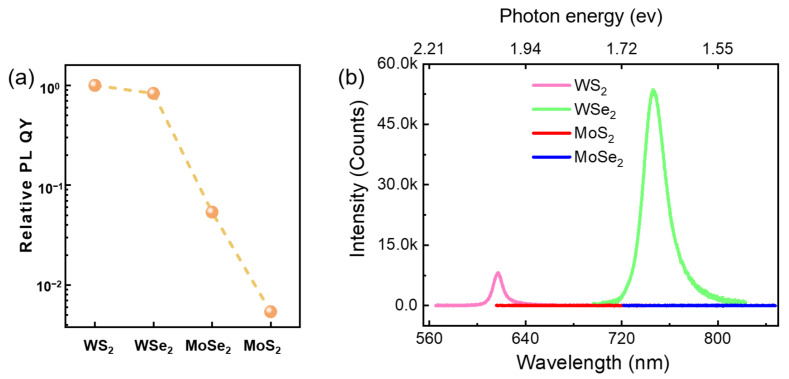
Relative PL QY and transient electroluminescence spectra of representative TMDC materials. (**a**) Relative PL QYs of four representative TMDC materials (WS_2_, WSe_2_, MoS_2_, MoSe_2_). (**b**) Transient EL spectra of the four representative TMDC-based devices driven by a square-wave voltage (square-wave voltage amplitude = ±9 V, frequency = 7 MHz). The lower and upper x-axes represent wavelength and photon energy, respectively.

Figure 1a shows an optical image of the AC gate-driven transient EL device based on monolayer WSe_2_ on a heavily doped silicon substrate with a 100 nm thick SiO_2_ layer gate oxide. Details on the fabrication process of the transient EL device are provided in the Methods Section. The WSe_2_ flake was contacted with a single metal electrode (source), and a bipolar square-wave voltage was applied between the back gate and source electrode, as shown in the inset of Figure 1b. When a square-wave gate voltage (±9 V, 1 MHz) was applied, EL emission was observed near the source/WSe_2_ interface, and the corresponding emission spectrum is shown in the inset of Figure 1c. Figure 1b shows the frequency dependence of the integrated WSe_2_ EL intensity. The nearly linear increase in EL intensity with driving frequency is attributed to the fact that transient EL occurs only during gate-voltage switching. Figure 1c shows the voltage-amplitude dependence of the integrated EL intensity. EL emission is observed once the gate voltage exceeds the turn-on voltage of 6V, which is primarily determined by the material bandgap and the series resistance of the device. Figure 1d–f show the results of the spatially resolved EL measurements under different electrode-grounding configurations. In all cases, the EL intensity reaches a maximum near the grounded source electrode and rapidly decreases with increasing distance from the source electrode, which is consistent with the typical interfacial emission feature of transient EL. After confirming the typical transient EL behavior of the WSe_2_ device, transient EL devices based on other monolayer TMDC materials were fabricated using nearly the same procedure. Figure 2b presents a comparison of the transient EL spectra obtained from different monolayer TMDC devices with the same device architecture and under identical driving conditions. The WSe_2_ device exhibits the strongest transient EL emission, whereas the WS_2_ device shows a weak EL signal. In contrast, no obvious EL emission is observed from the MoSe_2_ and MoS_2_ devices. This result reveals a pronounced material-dependent transient EL behavior. It should be noted that the absolute intensity of transient EL can be affected by multiple factors, including the intrinsic radiative efficiency of the material, defect-related nonradiative recombination, excitonic structure, contact resistance, Schottky barriers, and interfacial trap states. Therefore, the material-dependent EL behavior observed in Figure 2b cannot be directly attributed to a single factor. PLQY (photoluminescence quantum yield) provides a useful optical metric for evaluating the overall light-emission efficiency of a material under photoexcitation, reflecting the combined effects of radiative recombination, defect-related nonradiative losses, excitonic structure, and other material-dependent factors. To further analyze the possible origin of the material-dependent EL behavior, we first compared the PLQY values of four representative monolayer TMDCs, including WS_2_, WSe_2_, MoSe_2_, and MoS_2_, to assess their capability to convert photoexcited electron–hole pairs into photons. Direct measurement of the absolute PL QY is challenging. Nevertheless, relative PLQY values can be estimated from the instrument-response-corrected PL spectra and absorption spectra. Because the integrated PL intensity I is proportional to the product of the absorption coefficient at the excitation wavelength, $\alpha \left(\lambda_{exc}\right)$, and the PLQY, the relative PLQY can be estimated from $I/\alpha \left(\lambda_{exc}\right)$. Figure S1 presents the corrected PL spectra of monolayer WS_2_, WSe_2_, MoSe_2_, and MoS_2_. The PL spectrum of MoS_2_ was acquired with an integration time of 10 s, whereas the other spectra were recorded with an integration time of 0.1 s. Based on the absorption of these four materials at 532 nm (WS_2_, ~1.4%; WSe_2_, ~2.3%; MoSe_2_, ~3.7%; and MoS_2_, ~2.1%) [42], the relative PLQY values were estimated, as summarized in Figure 2a. Although WSe_2_ does not exhibit a higher relative PLQY than WS_2_, the WSe_2_ device shows a much stronger transient EL signal under the same device architecture and driving conditions (Figure 2b; square-wave voltage amplitude = ±9 V, frequency = 7 MHz). This comparison indicates that the stronger transient EL from WSe_2_ cannot be simply ascribed to intrinsically higher photoluminescence efficiency or lower nonradiative recombination loss.

**Figure 3:**
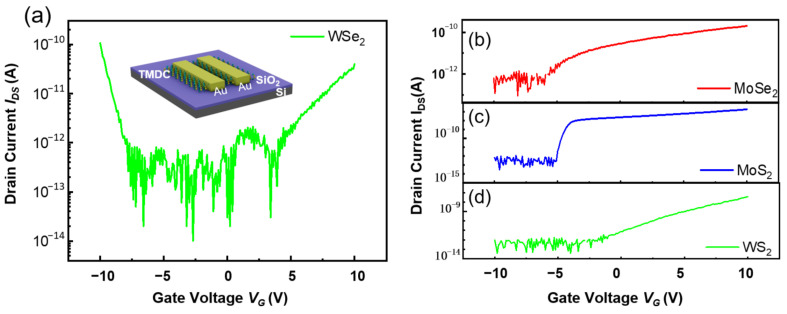
Transfer characteristics of representative TMDC devices. (**a**) Transfer characteristic of the WSe_2_ device. (**b**–**d**) Transfer characteristics of the MoSe_2_ (**b**), MoS_2_ (**c**), and WS_2_ (**d**) devices, respectively.

In transient EL devices, light emission occurs during the rapid switching of the AC gate voltage. When the gate voltage undergoes an abrupt change, the electric field across the device capacitance, namely the SiO_2_ gate dielectric, cannot respond instantaneously. As a result, the applied voltage is mainly dropped across the metal–semiconductor interface. The resulting transient band bending near the metal/TMDC interface induces interfacial carrier tunneling and local carrier redistribution, and the EL emission arises from radiative recombination of electrons and holes near the interface [33]. Accordingly, the transient EL intensity is related not only to the intrinsic emission capability of the material, which is partially reflected by its PLQY under photoexcitation, but also to whether electrons and holes can be supplied simultaneously near the metal/TMDC interface within the short time window after abrupt gate-voltage switching. Considering that the initial carrier population established under steady gate bias may influence the interfacial electron–hole distribution at the moment of abrupt gate-voltage switching, transfer-curve measurements were performed to assess the gate-dependent carrier states of the four TMDCs. The electrical transport properties of the four materials were systematically characterized based on field-effect transistor (FET) configurations, where Cr/Au (5 nm/60 nm) contacts were used as the electrodes. As the gate voltage V_G_ increases, the channel currents of WS_2_, MoS_2_, and MoSe_2_ increase monotonically, as clearly shown by the transfer curves in Figure 3b–d, indicating the n-type character of these three materials. The source-drain current (I_DS_) of WSe_2_ increases under both positive and negative gate biases, explicitly manifesting its ambipolar nature. Further, the carrier density of the four TMDCs under positive and negative gate biases was estimated by Equation (1) [43,44].

(1) $$n=q^{-1}C_{g}\left|V_{TH}-V_{G}\right|$$

where q denotes the charge of a single electron (or hole), C_g_ is the gate capacitance, V_TH_ is the threshold voltage, and V_G_ is the gate voltage. At a typical V_G_ = 9 V, the electron densities in WS_2_, MoS_2_, and MoSe_2_ are $2.63\times 10^{12}$, $3.02\times 10^{12}$, and $3.20\times 10^{12}$, respectively; at V_G_ = −9 V, the holes in all three materials are depleted. For WSe_2_, the electron and hole densities at V_G_ = ±9 V are $2.34\times 10^{11}$ and $1.07\times 10^{12}$, respectively.

**Figure 4:**
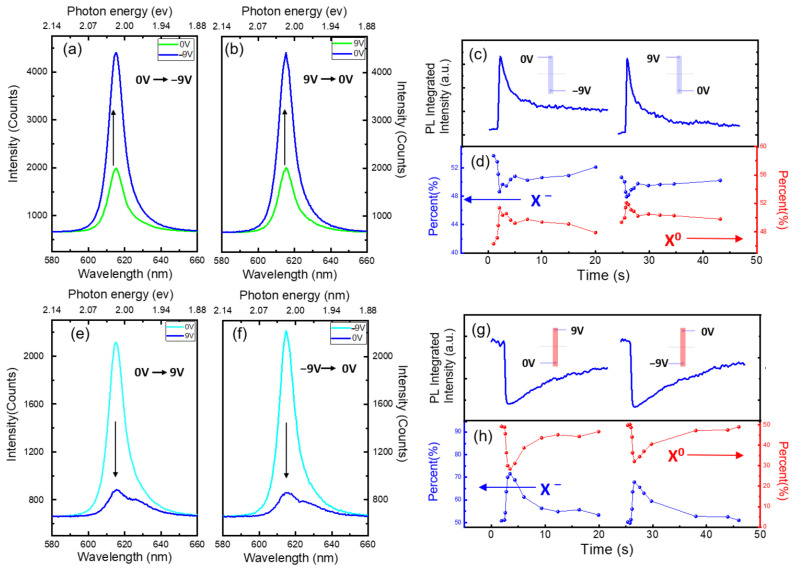
Time-resolved PL response of WS_2_ near the metal/WS_2_ interface under abrupt gate-voltage switching. (**a**,**b**) Changes in the WS_2_ PL spectra near the metal/WS_2_ interface before and after the abrupt decrease in gate voltage. The lower and upper x-axes represent wavelength and photon energy, respectively. (**c**) Corresponding temporal evolution of the integrated PL intensity after the abrupt decrease in gate voltage. (**d**) Time-dependent fractions of trion and neutral-exciton emission after the abrupt decrease in gate voltage. (**e**,**f**) Changes in the WS_2_ PL spectra near the metal/WS_2_ interface before and after the abrupt increase in gate voltage. (**g**) Corresponding temporal evolution of the integrated PL intensity after the abrupt increase in gate voltage. (**h**) Time-dependent fractions of trion and neutral-exciton emission after the abrupt increase in gate voltage.

Subsequently, micro-PL (μ-PL) measurements near the TMDC/Au interface under gate-voltage modulation were performed to probe the interfacial carrier-tunneling process induced by abrupt gate-voltage switching. The PL intensity near the TMDC/Au interface was monitored under low-frequency square-wave gate modulation. When the gate voltage changes abruptly, the electric field across the SiO_2_ gate dielectric cannot respond instantaneously, and a large transient voltage drop appears near the metal–semiconductor interface. This transient voltage drop can induce steep band bending at the Schottky contact and modulate the local optical response. For WS_2_, when the gate voltage is switched from 0 to −9 V or from 9 to 0 V, PL enhancement is instead observed in the n-type semiconductor WS_2_ (Figure 4a,b). This behavior cannot be explained solely by the electric-field-induced reduction in exciton oscillator strength, because such an effect would normally weaken PL. Instead, it indicates that carrier transfer at the metal–semiconductor interface likely plays an important role. Under an abrupt negative gate-voltage step (ΔV < 0), the upward band bending facilitates hole injection into WS_2_ while pushing electrons away from the interface, both of which reduce the excess electron concentration near the interface and thereby suppress trion formation. Given that charged excitons generally exhibit a lower quantum yield than neutral excitons [45,46], the suppression of trion formation will lead to PL enhancement. Meanwhile, the injected holes can also recombine with the remaining electrons to form neutral excitons, further enhancing the PL. Consistent with this interpretation, the PL enhancement after the negative gate-voltage step is accompanied by a sharp decrease in the trion fraction (Figure 4a,b,d). Figure 4c,d show the temporal evolution of the integral PL intensity and the trion/neutral-exciton fractions, respectively, after abrupt gate-voltage switching. As time evolves, the trion fraction gradually increases and the neutral-exciton fraction decreases, indicating that the initial interfacial tunneling process is followed by field-driven carrier migration and redistribution until a new steady state is established. When the gate voltage is switched from 0 to 9 V or from −9 to 0 V, the large voltage drops across the WS_2_/Au interface give rise to downward band bending [33]. This downward band bending promotes electron injection into WS_2_ while driving holes out of WS_2_, thereby further increasing the relative electron concentration and resulting in an enhanced trion fraction together with a reduced PL intensity. Figure 4e,f show the PL quenching after the positive gate-voltage step, while Figure 4g,h present the temporal evolution of the integral PL intensity and the trion/exciton fractions after this gate-voltage step. With increasing time, the neutral-exciton fraction gradually increases, accompanied by a corresponding decrease in the trion fraction. This evolution is attributed to initial interfacial carrier tunneling and subsequent field-driven carrier redistribution in WS_2_.

**Figure 5:**
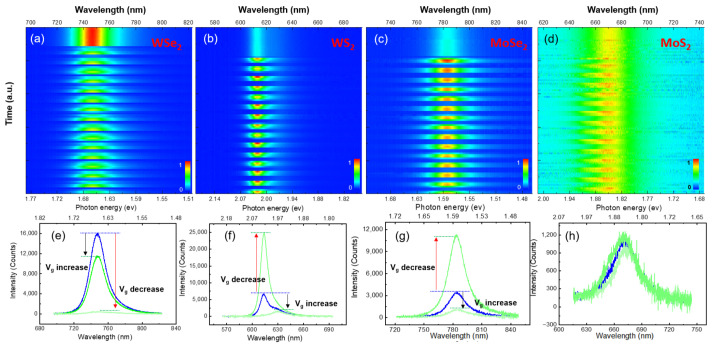
Gate-voltage-modulated PL dynamics of representative TMDC materials. (**a**–**d**) Two-dimensional time-resolved PL spectral maps of WSe_2_, WS_2_, MoSe_2_, and MoS_2_ acquired near the metal/TMDC interfaces under low-frequency square-wave gate-voltage modulation. (**e**–**h**) PL spectral changes near the metal/TMDC interfaces before and after abrupt gate-voltage switching. All spectra in Figure 5 are presented with wavelengths and the corresponding photon-energy axes.

Figure S2 presents the PL intensity variation near the metal/WSe_2_ interface after abrupt gate-voltage switching, together with the temporal evolution of the integrated PL intensity. When the gate voltage was switched from 0 to −9 V or from 0 to 9 V, PL quenching was observed in the ambipolar semiconductor WSe_2_ (Figure S2a,b). This PL quenching is attributed to carrier injection induced by abrupt gate-voltage switching. Specifically, when the gate voltage was switched from 0 to −9 V or from 0 to 9 V, holes or electrons were injected from the metal/WSe_2_ interface into WSe_2_, respectively. The rapid increase in the electron or hole concentration near the interface promotes positive- or negative-trion formation, respectively, leading to PL quenching. When the gate voltage was switched from 9 to 0 V (or from −9 to 0 V), PL quenching was also observed in WSe_2_, as shown in Figure S2a,b. This result is attributed to the simultaneous occurrence of carrier injection and the rapid extraction of electrostatically accumulated carriers during abrupt gate-voltage switching. In these processes, carrier injection and the carrier extraction jointly increase the relative electron or hole concentration near the interface, thereby promoting negative/positive trion formation and reducing the PL intensity. Notably, WSe_2_ exhibits more pronounced PL quenching when the gate voltage is switched from 0 V to −9 V or from 9 V to 0 V, which corresponds to the hole-injection process, as indicated by the red arrows in Figure S2a,b. The stronger response is likely associated with the formation of a p-type contact at the metal/WSe_2_ interface. The reduced hole-injection barrier enables more efficient hole injection, leading to stronger modulation of interfacial carrier population and PL intensity. Then, we performed gate-voltage-modulated PL measurements on four TMDC materials, namely n-type WS_2_, n-type MoSe_2_, n-type MoS_2,_ and ambipolar WSe_2_, and examined the temporal evolution of their PL spectra. Figure 5a–d present two-dimensional time-resolved PL spectral maps near the metal/TMDC interfaces under low-frequency square-wave gate-voltage modulation, acquired with a CCD camera. WSe_2_ exhibits PL quenching under both positive and negative gate-voltage steps, whereas the n-type semiconductors WS_2_ and MoSe_2_ show PL quenching under positive gate-voltage steps but PL enhancement under negative gate-voltage steps (see Figure 5e–g). The absence of pronounced PL modulation in MoS_2_ is likely associated with its relatively low quantum efficiency, which makes its PL intensity less sensitive to gate-voltage-induced carrier variation (see Figure 5h). These observations support the occurrence of gate-voltage-switching-induced interfacial carrier tunneling. Specifically, the transient interfacial electric field generated by abrupt gate-voltage switching drives carrier tunneling at the metal/TMDC interface and modulates the local carrier distribution, which is reflected in the time-dependent PL response. Therefore, the gate-voltage-modulated PL results provide optical evidence for interfacial carrier modulation during gate-voltage switching. Based on the initial carrier distribution and interfacial tunneling process, the transient relative electron–hole concentrations near the metal/TMDC interface under alternating electric-field driving can be further analyzed. The metal–semiconductor interfaces of n-type WS_2_ and MoSe_2_ tend to form locally electron-rich carrier states after abrupt gate-voltage switching, which favors trion formation and suppresses efficient radiative recombination. In contrast, ambipolar WSe_2_ can accumulate electrons and holes near the metal/semiconductor interface under positive and negative gate voltages, respectively, leading to a more balanced interfacial electron–hole population after abrupt gate-voltage switching. This balanced carrier distribution favors radiative recombination and gives rise to stronger transient electroluminescence. The detailed analysis is provided in Supplementary Note S1, and the corresponding schematic illustration of transient interfacial carrier redistribution and corresponding local energy-alignment diagrams at the metal/TMDC interfaces during gate-voltage switching are shown in Figure S3. Overall, this polarity-dependent interfacial carrier-distribution picture offers a reasonable explanation for the experimentally observed stronger transient EL from ambipolar WSe_2_ devices, suggesting that carrier transport polarity may be an important factor affecting the material-dependent EL intensity.

Based on the above results, we cautiously propose that the stronger transient EL observed in WSe_2_ is more likely associated with its ambipolar carrier transport characteristics. The relative PLQY measurements show that the stronger EL from WSe_2_ cannot be simply attributed to more efficient conversion of electron–hole pairs into photons. Meanwhile, the transfer curves and gate-modulated PL results indicate that n-type WS_2_ is more likely to form a single-carrier-dominated local nonequilibrium state during gate-voltage switching, whereas ambipolar WSe_2_ is more likely to establish a relatively balanced transient electron–hole local distribution near the interface. Because transient EL occurs within a short time window after abrupt voltage switching, its radiative recombination efficiency depends not only on the injection strength of a single carrier type but also on whether electrons and holes can be supplied simultaneously near the interface. For n-type WS_2_, the single-carrier-dominated interfacial carrier environment can limit electron–hole radiative recombination and may promote charged-exciton formation, thereby weakening transient EL. In contrast, the more balanced transient electron and hole populations near the metal/WSe_2_ interface are more favorable for neutral-exciton formation and radiative recombination. Therefore, the above polarity-dependent carrier-distribution picture is consistent with the experimentally observed weaker transient EL from WS_2_ devices and stronger transient EL from WSe_2_ devices under the same device structure and driving conditions, which provides a reasonable explanation for the material-dependent transient EL behavior. It should be noted that contact properties, Schottky barriers, and interface states may still affect the absolute EL intensity of specific devices, and their influence cannot be completely excluded. However, in transient EL devices, abrupt gate-voltage switching can induce a large transient voltage drop and steep band bending near the metal/TMDC interface, thereby driving transient carrier tunneling. Consequently, transient EL is expected to depend relatively weakly on contact properties. For example, Lien et al. showed that different metal contacts can cause orders-of-magnitude changes in the transistor on-current, whereas the corresponding integrated transient EL intensity changes only by a few-fold factor [33]. This suggests that the approximately 15-fold difference (obtained from the ratio of their integrated EL intensities) in integrated EL intensity between WS_2_ and WSe_2_ may not primarily arise from contact-characteristic differences. Taken together, these considerations support the view that the polarity-dependent transient carrier distribution near the metal/TMDC interface may play a more important role in the stronger transient EL observed in WSe_2_, although possible contributions from contact characteristics cannot be fully excluded.

## 4. Conclusions

In summary, we investigated the influence of transport carrier polarity on the transient electroluminescence behavior of two-dimensional TMDC devices. By comparing TMDC materials with different transport polarities, we found that ambipolar WSe_2_ exhibits a much stronger transient EL signal than n-type WS_2_, suggesting that transport polarity may be one of the important material factors affecting transient EL emission. To further understand this behavior, we compared the relative PLQY values of different materials and combined transfer characteristics with gate-modulated PL measurements to analyze the carrier accumulation/depletion states under different gate voltages, as well as the spectral responses associated with interfacial carrier tunneling during abrupt gate-voltage switching. Based on these results, we propose a possible physical picture: in n-type TMDC materials, gate-voltage switching is more likely to generate an electron-dominated nonequilibrium carrier distribution near the interface, which is unfavorable for efficient electron–hole radiative recombination. In contrast, ambipolar WSe_2_ can support electron and hole accumulation under positive and negative gate voltages, respectively, and is therefore more likely to form a relatively balanced interfacial electron–hole population after gate-voltage switching, leading to stronger transient EL. It should be noted that, given the relatively weak dependence of transient EL on contact properties, the approximately 15-fold difference in EL intensity observed between WS_2_ and WSe_2_ is unlikely to mainly originate from differences in contact characteristics. Nevertheless, contributions from contact properties and other material-related factors to the absolute EL intensity cannot be excluded. This work indicates that semiconductor transport polarity may serve as an important material parameter to consider when optimizing low-dimensional transient electroluminescent devices.

## Data Availability

Data underlying the results presented in this paper are not publicly available at this time but may be obtained from the authors upon reasonable request.

## Supplementary Materials

The following supporting information can be downloaded at: https://www.mdpi.com/article/10.3390/nano16130827/s1, Figure S1: The corrected PL spectra of monolayer WS_2_, WSe_2_, MoSe_2_, and MoS_2_. Figure S2: Time-resolved PL response of WSe_2_ near the metal/WSe_2_ interface under abrupt gate-voltage switching. Figure S3: Schematic illustration of transient interfacial carrier redistribution and corresponding local energy-alignment diagrams at the metal/TMDC interfaces during gate-voltage switching. Figure S4: AFM images and corresponding height profiles of four representative TMDC materials. Supplementary Note S1: Qualitative analysis of transient interfacial carrier redistribution under AC gate-voltage driving.
