# Supplementary material for: Role of Transport Polarity in Transient Electroluminescence of Two-Dimensional TMDC Semiconductors
Source: Nanomaterials (Basel). 2026 Jul 6;16(13):827. doi: 10.3390/nano16130827 (PMC13362784; doi:10.3390/nano16130827)
Supplement: Supplementary file 1 [file nanomaterials-16-00827-s001.zip › nanomaterials-4371147-supplementary.pdf]

Supplementary Materials

Article

Role of Transport Polarity in Transient Electroluminescence of Two-Dimensional TMDC Semiconductors

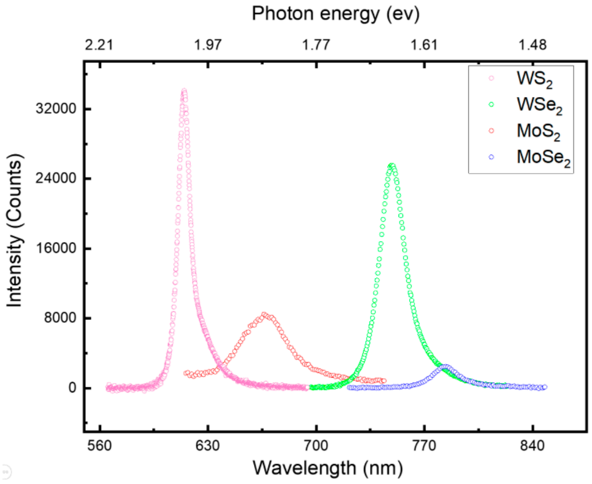

Figure S1. The corrected PL spectra of monolayer WS_2, WSe_2, $MoSe_2$, and MoS_2.

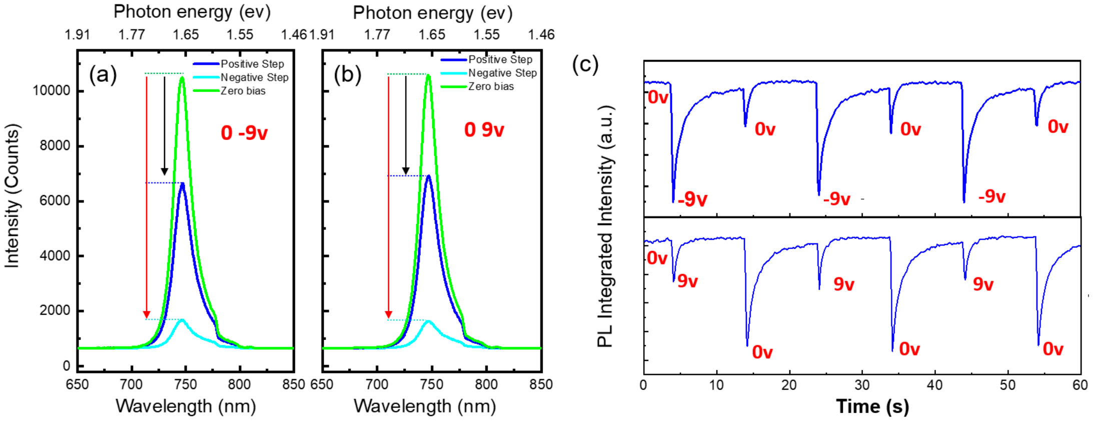

Figure S2. Time-resolved PL response of WSe_2 near the metal/WSe_2 interface under abrupt gate-voltage switching. (**a, b**) Changes in the WSe_2 PL spectra near the metal/WSe_2 interface before and after abrupt gate-voltage switching, where positive and negative steps correspond to $\Delta V > 0$ and $\Delta V < 0$, respectively. The lower and upper x-axes represent wavelength and photon energy, respectively. (**c**) Corresponding temporal evolution of the integrated PL intensity after abrupt gate-voltage switching.

Supplementary Note S1. Qualitative analysis of transient interfacial carrier redistribution under AC gate-voltage driving

The transient electroluminescence (EL) process is closely related to the carrier population established near the metal/TMDC interface before gate-voltage transition and to the subsequent interfacial carrier redistribution induced by abrupt gate-voltage switching. Based on the transfer characteristics discussed in the main text, ambipolar WSe_2 can be electrostatically driven into electron- and hole-rich states under positive and negative gate voltages, respectively. In contrast, n-type WS_2 and $MoSe_2$ mainly evolve between electron-accumulated and electron-depleted states within the accessible gate-voltage range. The gate-modulated PL results further show that abrupt gate-voltage switching induces distinct local PL responses near the metal/ TMDC interfaces, providing optical evidence for polarity-dependent interfacial carrier modulation. On the basis of these observations, we propose the qualitative carrier-redistribution picture illustrated in Fig. S3. For ambipolar WSe_2, switching the gate voltage from 9 V to -9 V generates a transient interfacial electric field that drives hole injection into WSe_2. Because the pre-existing electron density near the interface is lower than that in n-type WS_2 or $MoSe_2$ under V_G=9 V, the injected holes can produce a more balanced interfacial electron–hole population than in the n-type materials. This more balanced carrier distribution favors radiative recombination and suppresses trion-dominated recombination (see Fig. S3b and Fig. S3d). Similarly, switching the gate voltage from −9 V to 9 V drives electron injection into WSe_2. The pre-accumulated holes allow the injected electrons to form a relatively high and balanced interfacial electron–hole population, enabling more efficient radiative recombination (see Fig. S3a and Fig. S3c). For n-type WS_2 and $MoSe_2$, switching the gate voltage from 9 V to −9 V drives hole injection into the materials. Because the pre-existing electron density near the interface is higher than that in ambipolar WSe_2, the interfacial carrier distribution remains more electron-rich than that in WSe_2 (see Fig. S3d and Fig. S3b). This leads to a less balanced electron–hole population, thereby limiting radiative recombination and weakening the electroluminescence intensity under the negative gate-voltage step. When the gate voltage is switched from −9 V to 9 V, electrons are injected into the n-type materials. The low initial hole density near the interface leads to a strong electron-dominated carrier imbalance after electron injection, which significantly reduces radiative recombination under the positive gate-voltage step (see Fig. S3c).

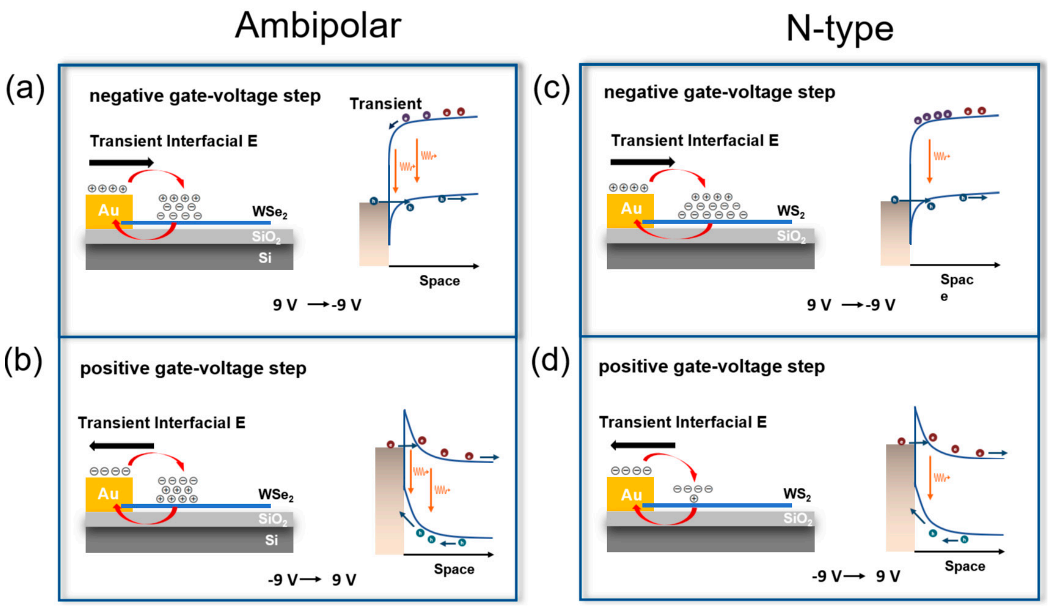

Figure S3. Schematic illustration of transient interfacial carrier redistribution and corresponding local energy-alignment diagrams at the metal/TMDC interfaces during gate-voltage switching. (a, b) Carrier redistribution and corresponding local energy-alignment diagrams near the metal/WSe_2 interface under negative and positive gate-voltage steps. (c, d) Carrier redistribution and corresponding local energy-alignment diagrams near the metal/WS_2 interface under negative and positive gate-voltage steps.

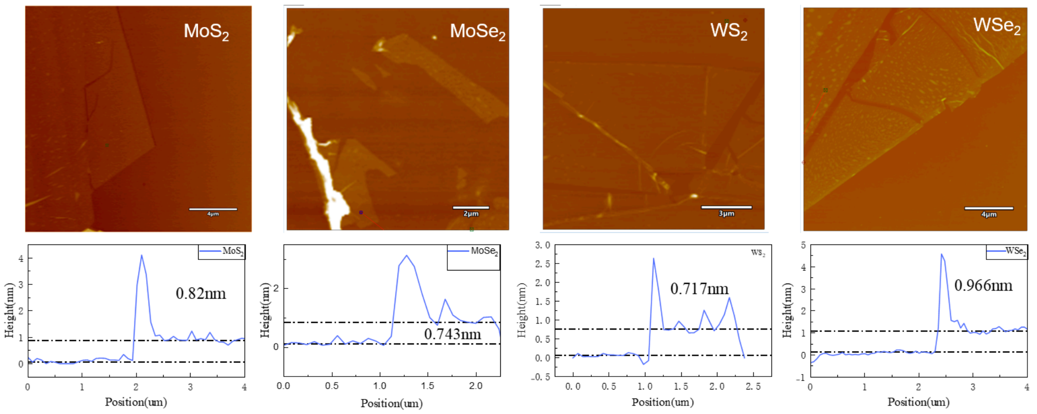

Figure S4. AFM images and corresponding height profiles of four representative TMDC materials.
